# Degradation reduces the diversity of nitrogen-fixing bacteria in the alpine wetland on the Qinghai-Tibet Plateau

**DOI:** 10.3389/fpls.2022.939762

**Published:** 2022-08-04

**Authors:** Chengyi Li, Xilai Li, Yuanwu Yang, Yan Shi, Honglin Li

**Affiliations:** ^1^College of Agriculture and Animal Husbandry, Qinghai University, Xining, China; ^2^State Key Laboratory of Plateau Ecology and Agriculture, Qinghai University, Xining, China; ^3^School of Environment, The University of Auckland, Auckland, New Zealand

**Keywords:** Qinghai-Tibet Plateau, alpine wetland, wetland succession, *nifH* gene, nitrogen-fixing bacteria

## Abstract

Biological nitrogen fixation is a key process in the nitrogen cycle and the main source of soil available nitrogen. The number and diversity of nitrogen-fixing bacteria directly reflect the efficiency of soil nitrogen fixation. The alpine wetland on the Qinghai-Tibet Plateau (QTP) is degrading increasingly, with a succession toward alpine meadows. Significant changes in soil physicochemical properties accompany this process. However, it is unclear how does the soil nitrogen-fixing bacteria change during the degradation processes, and what is the relationship between these changes and soil physicochemical properties. In this study, the *nifH* gene was used as a molecular marker to further investigate the diversity of nitrogen-fixing bacteria at different stages of degradation (none, light, and severe degeneration) in the alpine wetland. The results showed that wetland degradation significantly reduced the diversity, altered the community composition of nitrogen-fixing bacteria, decreased the relative abundance of Proteobacteria, and increased the relative abundance of Actinobacteria. In addition to the dominant phylum, the class, order, family, and genus of nitrogen-fixing bacteria had significant changes in relative abundance. Analysis of Mantel test showed that most soil factors (such as pH, soil water content (SWC), the organic carbon (TOC), total nitrogen (TN), and soil C:P ratio) and abundance had a significant positive correlation. TOC, TN, total phosphorus (TP), soil C:P ratio and Shannon had a significant positive correlation with each other. The RDA ranking further revealed that TOC, SWC, and TN were the main environmental factors influencing the community composition of nitrogen-fixing bacteria. It is found that the degradation of the alpine wetland inhibited the growth of nitrogen-fixing bacteria to a certain extent, leading to the decline of their nitrogen-fixing function.

## Introduction

The nitrogen cycle is one of the globally important biogeochemical processes that are almost entirely mediated by microorganisms in the environment (Wang et al., 2015). As nitrogen fixation is a key process in the microbial nitrogen cycle (Levy-Booth et al., 2014), biological nitrogen fixation reduces atmospheric unavailable N_2_ to ${\text{NH}}_{4}^{+}$ that can be absorbed and utilized by plants (Simon et al., 2014). Globally, ~198 Tg of nitrogen per year is fixed by bacteria in natural terrestrial and aquatic ecosystems (Fowler et al., 2018). The fixed nitrogen can alleviate nitrogen constraints on the function of the forest (Deluca et al., 2002), grassland (Regan et al., 2016), desert (Ramond et al., 2018), and marine ecosystems (Zehr, 2011). Therefore, biological nitrogen fixation represents the largest input of non-anthropogenic nitrogen intake to most terrestrial and aquatic ecosystems (Vitousek et al., 1997; Paul et al., 2010), and is also considered to be the largest source of reactive nitrogen on Earth (Cleveland et al., 1999; Galloway et al., 2004). All nitrogen-fixing bacteria discovered so far belong to prokaryotes, and there is a catalytic nitrogenous in these nitrogen-fixing bacteria (Kang et al., 2013). Nitrogenous complexes are composed of MoFeprotein encoded by the *nifD* and *nifK* genes and ferritin encoded by the *nifH* genes (Zehr et al., 2003). The *nifH* gene exists only in nitrogen-fixing bacteria, and its phylogenetic relationship is consistent with 16S rRNA, which is a genetic marker of nitrogen-fixing bacteria (Kang et al., 2013). It is commonly used to prove the existence of nitrogen-fixing bacteria and to reveal the relationship between the community structure of nitrogen-fixing bacteria and the environment (Liu et al., 2017). In addition, the abundance, diversity, and community composition of nitrogen-fixing bacteria have been considered as the key biological factors to determine the nitrogen-fixing capacity of the soil (Lindsay et al., 2010; Reed et al., 2010; Stewart et al., 2011). Therefore, determining them can help to understand the nitrogen cycle in terrestrial ecosystems.

With an average altitude of over 4,000 m, the Qinghai-Tibet Plateau (QTP) is known as the “roof of the world” and the “third pole.” It is an important ecological security barrier for China and even Asia, with important functions such as carbon and nitrogen storage, water recharge, and climate regulation (Sun et al., 2012). Its unique geographical location and climatic conditions have nurtured the alpine ecosystems that are sensitive to climate change and human activities (Zhao and Shi, 2020), including grasslands, wetlands, deserts, and forests. Among them, the alpine wetlands cover an area of over 1 × 10^5^ km^2^, accounting for nearly 30% of China's wetland area. They are widely distributed in the main region of the QTP, including the headwater of three rivers (Sanjiangyuan region) in China (Xing et al., 2009; Zhao et al., 2014; Zhao and Shi, 2020). Over the past few decades, due to the influence of human activities (drainage and overgrazing), the groundwater level of the alpine wetlands has decreased, and vegetation communities have changed (Straková et al., 2010; Munir et al., 2014; Wu et al., 2021). In addition, global climate change has further exacerbated the human-induced groundwater level decline of the alpine wetlands (Liu et al., 2019). Lower groundwater levels promote the decomposition of organic matter and loss of humus and peat layers in soil (Shuang et al., 2009; Lin et al., 2021), and may simultaneously promote the conversion of soil nitrogen to aerobic processes (Espenberg et al., 2018), resulting in soil nitrogen reduction (Liu L. et al., 2018). The typical regression succession pattern of the alpine wetland is a marsh (intact wetland) → marsh meadow (early degradation stage) → alpine meadow (degraded) (Guo et al., 2013; Luan et al., 2014). The development process of wetland has been transformed into humification and the REDOX process of meadow soil (Yang and Wang, 2001; Liang et al., 2007). It has been experimentally proven that soil microbial communities are more sensitive to environmental changes than plants and animals (Qian and Ricklefs, 2004; Zhou et al., 2008; Zhang et al., 2013). Soil microorganisms are the medium for all soil ecological functions, and their community composition and diversity are key to studying soil ecological balance and soil ecological functions (Shanmugam et al., 2017). Therefore, it is of great scientific importance to study the structure and diversity of soil microbial communities in the alpine wetlands in response to the alpine wetland degradation.

The degradation of the alpine wetland has a significant impact on soil microbial community structure and diversity (Li et al., 2021). However, different microbial taxa show different ecological functions in the biogeochemical cycle of elements, and the specific microbial community structure can affect a variety of ecosystem processes (Allison et al., 2013). At present, there are relatively few studies on the impact of the alpine wetland degradation on soil nitrogen-fixing bacteria community structure, only found by Wu et al. (2016), which reported that nitrogen cycling (nitrogen-fixing, ammonia oxidation, and denitrification), microbial community structure, and relative abundance changed due to the alpine wetland degradation. Even within the same type of ecosystem, soil nitrogen-fixing bacteria abundance, diversity, and community composition varied considerably among the study sites (Che et al., 2017; Wang et al., 2017). So far, many studies have confirmed that complex interactions exist among plant communities, soil physics and chemistry, and soil microorganisms (Tedersoo et al., 2014; Delgado-Baquerizo et al., 2018). The degradation of the alpine wetland is accompanied by significant changes in soil physicochemical properties (Li et al., 2021). The relationship between soil nitrogen-fixing bacteria and soil physicochemical properties during this process is not yet clear. Therefore, studying the changes in nitrogen-fixing bacteria composition and its relationship with the soil environment during the alpine wetland degradation can help us understand the soil microorganisms changing mechanisms and nitrogen-cycling processes of the alpine wetland under human disturbance and climate change.

In this study, none, lightly, and severely degraded alpine wetlands in the source region of the Yellow River on the QTP were selected as research objects, and the diversity of nitrogen-fixing bacteria was identified by using the *nifH* gene as a molecular marker. To reveal the response of nitrogen-fixing bacteria diversity to the degradation of the alpine wetlands, explore the critical environmental factors that affect the community structure of nitrogen-fixing bacteria, and provide scientific theoretical support for the protection and management of the alpine wetlands, this study aims to: (1) explore the structure and composition of the nitrogen-fixing bacteria community in the alpine wetland and its various features in the degraded succession sequence and (2) access the correlation between the diversity of nitrogen-fixing bacteria and environmental factors.

## Materials and methods

### Description of sample points

**Figure 1:**
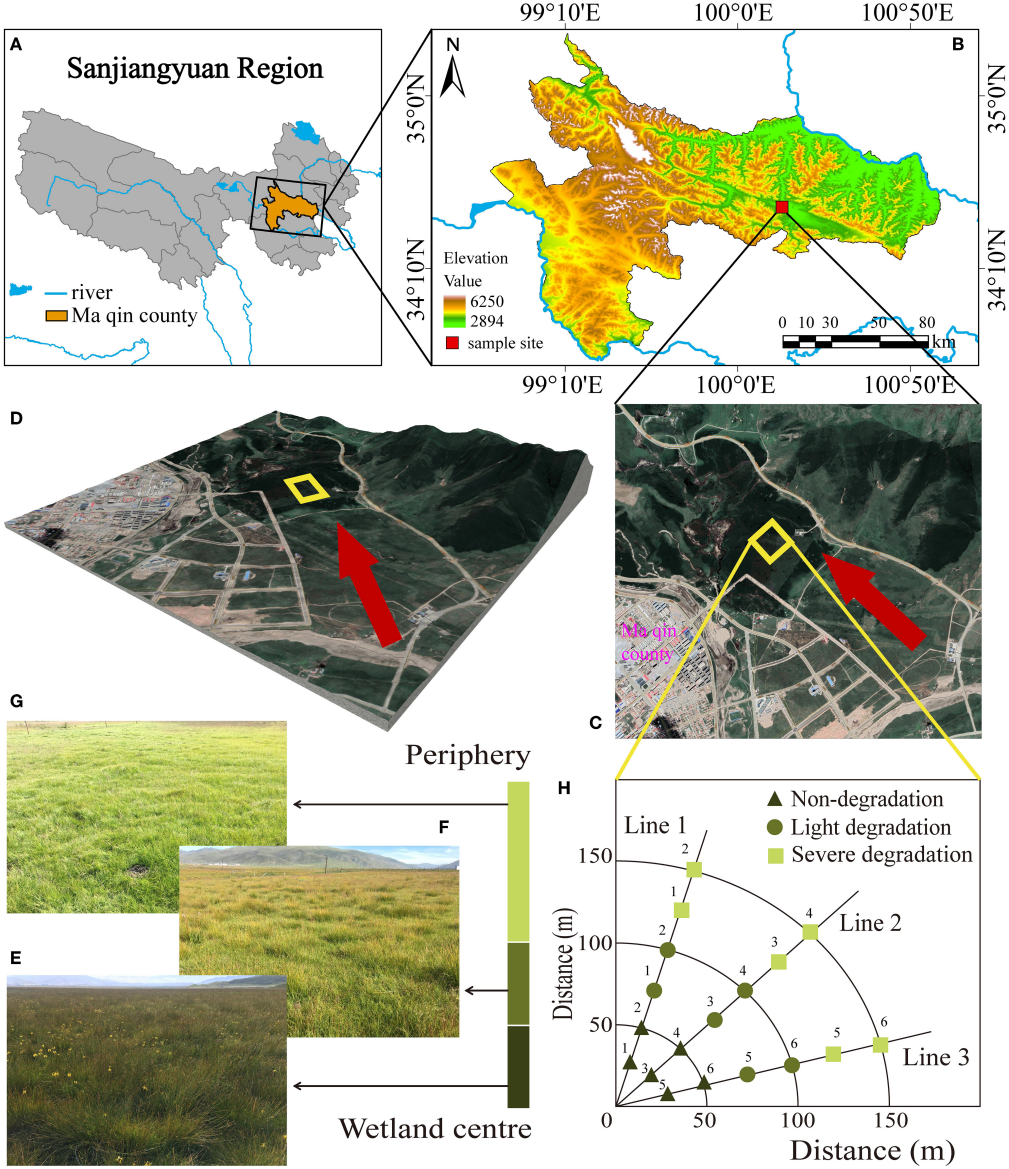
Location of the study site and the experiment sites and treatments. **(A)** Sanjiangyuan area, **(B)** the study area, **(C)** sampling site, **(D)** 3D view, **(E)** non-degraded the alpine wetland landscape, **(F)** light degraded the alpine wetland landscape, **(G)** severe degraded the alpine wetland landscape, and **(H)** the distribution of sampling points.

The study site is located in Dawu Town, Maqin County, Guoluo Tibetan Autonomous Prefecture, Qinghai Province, western China (34°27′ 48″ N, 100°12′ 49″ E, with an average altitude of 3,730 m) (Figures 1A,B). It has a continental cold and moist climate with an average annual temperature of −3.9 to −3.5°C and average annual precipitation of 423–565 mm (Lin et al., 2021). The alpine wetland in this area is rich in resources, and the plant communities in the experimental sites are mainly composed of *Cyperaceae* and *Gramineae*. The current land-use type is winter grazing. In this area, the wetland gradually changed to swamp meadows due to the increasing number and the decreasing size of freeze-thaw mounds, resulting in a severe degree of degradation. The characteristics of freeze-thaw banks in the alpine wetlands disappeared and transformed into alpine meadows. Dominant species, vegetation coverage, the average height of vegetation, number, and size of freeze-thaw mounds were recorded (Supplementary Table 1). The experimental area is 4 hectares (Figures 1C,D), and the selected sample plot extends outward from the alpine wetland by the sample line method. Three sample lines are randomly selected in different degradation stages (Non-degradation, ND, Figure 1E; Light degradation, LD, Figure 1F; Severe degradation, SD, Figure 1G) to collect soil samples. Each sampling line is sampled at 25 m intervals and tested six times in each location (Figure 1H).

### Soil sample collection

In August 2020, during the vigorous growth period of forage grass, according to the principles of “random,” “equal amount,” and “multi-point mixing” in 18 sample points, five sample plots with specifications of 10 × 10 cm were selected for sampling by five-point sampling method. Because surface soil is more prone to degradation than deep soil (Liu L. et al., 2018), we focused on the changes of nitrogen-fixing bacteria in shallow soil in this study. The sampling tool is disinfected with alcohol, and the ground of 0–20 cm soil layer with the exact weight is evenly mixed. After removing impurities, such as roots and stones, soil samples are put into a sterile tube by the “quartering method.” Only 10 g of each soil sample was used for detecting the *nifH* gene (tested by Beijing Allwegene Technology Co., Ltd). These sub-samples were temporarily stored in a liquid nitrogen tank in the field, and the tanks were put into chilly bins filled with dry ice for delivery. The remaining soil samples were taken back to the laboratory, and some fresh soil samples were stored in a low-temperature refrigerator at 4°C. In contrast, others were naturally dried and ground, and sieved to analyze soil's physical and chemical properties.

### Analysis and determination of soil samples

#### Physical and chemical properties

Soil moisture was measured by TDR350 produced by Spectrum Company (USA). The contents of total nitrogen (TN), total phosphorus (TP), available phosphorus (AP), ammonium nitrogen (${\text{NH}}_{4}^{+}$-N), and nitrate-nitrogen (NO${}_{3}^{-}$-N) were determined by the AA3 Continuous Flow Analyzer from SEAL company (Germany), and organic carbon content (TOC) was determined by external oxidation heating method of potassium dichromate-concentrated sulfuric acid solution. Soil pH was determined by a pHS-3C pH meter (soil: water = 1: 2.5). There were significant differences in soil physicochemical properties and stoichiometric ratio in the alpine wetlands at different degradation stages (Supplementary Table 2).

#### Illumina MiSeq sequencing *nifH* gene

The test steps are: genomic DNA extraction—genomic DNA quality inspection—PCR amplification—PCR product electrophoresis detection—PCR product purification—Miseq library construction—Miseq library quality inspection—Illumina Miseq machine sequencing platform.

##### DNA extraction

Soil microbiome DNA was extracted by the PowerSoil DNA Isolation Kit (MoBio Laboratories, Inc., CA) [Omega E.Z.N.A. Stool DNA Kit (Omega Bio-Tek, Inc., USA)]. The extracted DNA was assayed for DNA quality and concentration using a Nanodrop 2000 (ThermoFisher Scientific, Inc., USA). The samples were stored at −20°C for subsequent experiments.

##### PCR amplification

Gene primer for *nifH*-F: 5′-AAAGGYGGWATCGGYAARTCCACCAC-3′ and *nifH*-R: 5′-TTGTTSGCSGCRTACATSGCCATCAT-3′ (Rösch and Bothe, 2005). PCR reaction system (total system is 25 μL): 12.5 μL 2xTaq Plus Master Mix II (Vazyme Biotech Co., Ltd, China), 3 μL BSA (2 ng/μL), 1 μL Forward Primer (5 μM), 1 μL Reverse Reaction parameters: 95°C pre-denaturation for 5 min; denaturation at 95°C for 45 s, annealing at 55°C for 50 s, extension at 72°C for 45 s, 28 cycles; extension at 72°C for 10 min. The PCR products were amplified using 1% agarose gel electrophoresis to detect the size of the amplified target bands and purified using the Agencourt AMPure XP nucleic acid purification kit (Beckman Coulter, Inc., USA).

##### MiSeq sequencing

PCR products were used to construct microbial diversity sequencing libraries using the NEB Next Ultra II DNA Library Prep Kit (New England Biolabs, Inc., USA). Paired-end sequencing was performed at the Beijing Allwegene Technology Co., Ltd. using the Illumina Miseq PE300 high-throughput sequencing platform (Illumina, Inc., USA). The sequenced raw sequences were uploaded to the NCBI's SRA database.

##### Data processing

To make the analysis result more accurate and reliable, the off-board data is split by the QIIME (v1.8.0) software according to the Barcode sequence, and the data quality control is carried out by the Pear (v0.9.6) software. After sequence splicing, filtering, and chimerism removal, the optimized sequence is obtained, and then OTUs (operational taxonomic units) clustering and annotation are carried out. The UPARSE method was used to statistically analyze the biological information of OTUs at a 97% similarity level (Edgar, 2013). Based on the clustering results, diversity analysis can be carried out; OTUs were annotated with the database of Silva (Release 128/132 https://www.arb-silva.de/) (Quast et al., 2012). Based on the annotation results, the species information of each classification can be obtained. Then, the correlation analysis of sample composition and community results among samples can be carried out.

Shannon-Wiener is an index reflecting the microbial diversity of samples, and it is constructed by using the microbial diversity index of each sample at different sequencing depths to reflect the microbial diversity of each sample with different sequencing quantities (Quast et al., 2012). The curve tends to be flat, and the sequencing data was large enough to reflect most of the microbial information in the sample (Supplementary Figure 1).

### Data analysis

The differences in soil physicochemical properties, nitrogen-fixing bacteria diversity (Chao1, Observed_species, PD_whole_tree, and Shannon), and species abundance in different degradation stages were examined by the one-way ANOVA and multiple comparison (Duncan) analysis with the SPSS Statistics 20.0 statistical analysis software.

The overlap of the number of OTUs in different degradation stages was represented by the Venn diagram. In the R software environment, the differences of *nifH* gene communities in different degradation stages were visualized by principal coordinate analysis (PCoA). To determine whether the degradation of the alpine wetlands changed the community composition of the *nifH* gene, a permutation variance analysis (PERMANOVA) was carried out using the Bray-Curtis similarity index. The effect was analyzed by linear discriminant analysis (LEfSe; Score = 3). Biomarkers with significant differences in abundance among different degradation stages were identified.

The Venn diagram and species composition histogram were counted and plotted by R language, and the Shannon-Wiener index was analyzed by mothur based on OTUs clustering results. The correlation analysis between the Mantel test and Pearson was completed by R language ggplot2, ggcor, and dplyr software package. Conducts the Redundancy analysis (RDA) with Canoco 5.0.

## Results

### Diversity of nitrogen-fixing bacteria

**Figure 2:**
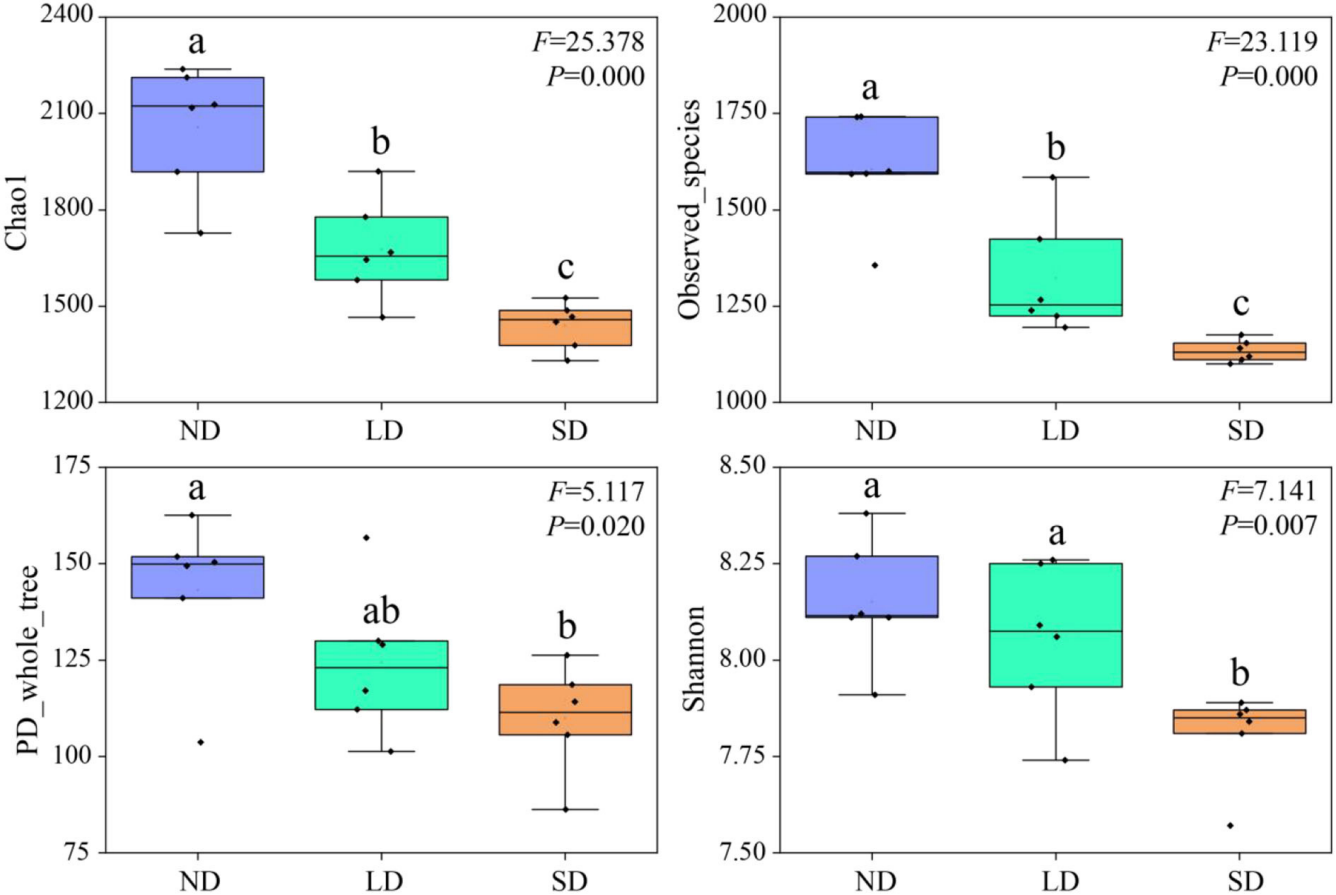
Abundance and diversity index of nitrogen-fixing bacteria community. ND is non-degradation and LN and SD are the light and severe degradation stages of the alpine wetland, respectively. The center mark in each box diagram represents the median, and the bottom and top edges of the box represent 25 and 75%, respectively. Different lowercase letters represent significant differences between different degradation stages.

Chao1, Observed_species, PD_whole_tree, and Shannon indexes decrease with the deterioration of the alpine wetland (Figure 2). Compared with ND, LD Chao1, and Observed_species indexes were significantly decreased by 18.51 and 17.57%, respectively (*P* < 0.05). SD Chao1, Observed_species, PD_whole_tree, and Shannon indexes were significantly reduced by 30.00, 29.33, 23.21, and 4.21%, respectively (*P* < 0.05). Compared with LD, SD Chao1, Observed_species, and Shannon indexes significantly decreased by 14.10, 14.26, and 3.08%, respectively (*P* < 0.05). These results indicate that the degradation of the alpine wetlands is able to decline the diversity of nitrogen-fixing bacteria significantly.

### Composition of nitrogen-fixing bacteria community

**Figure 3:**
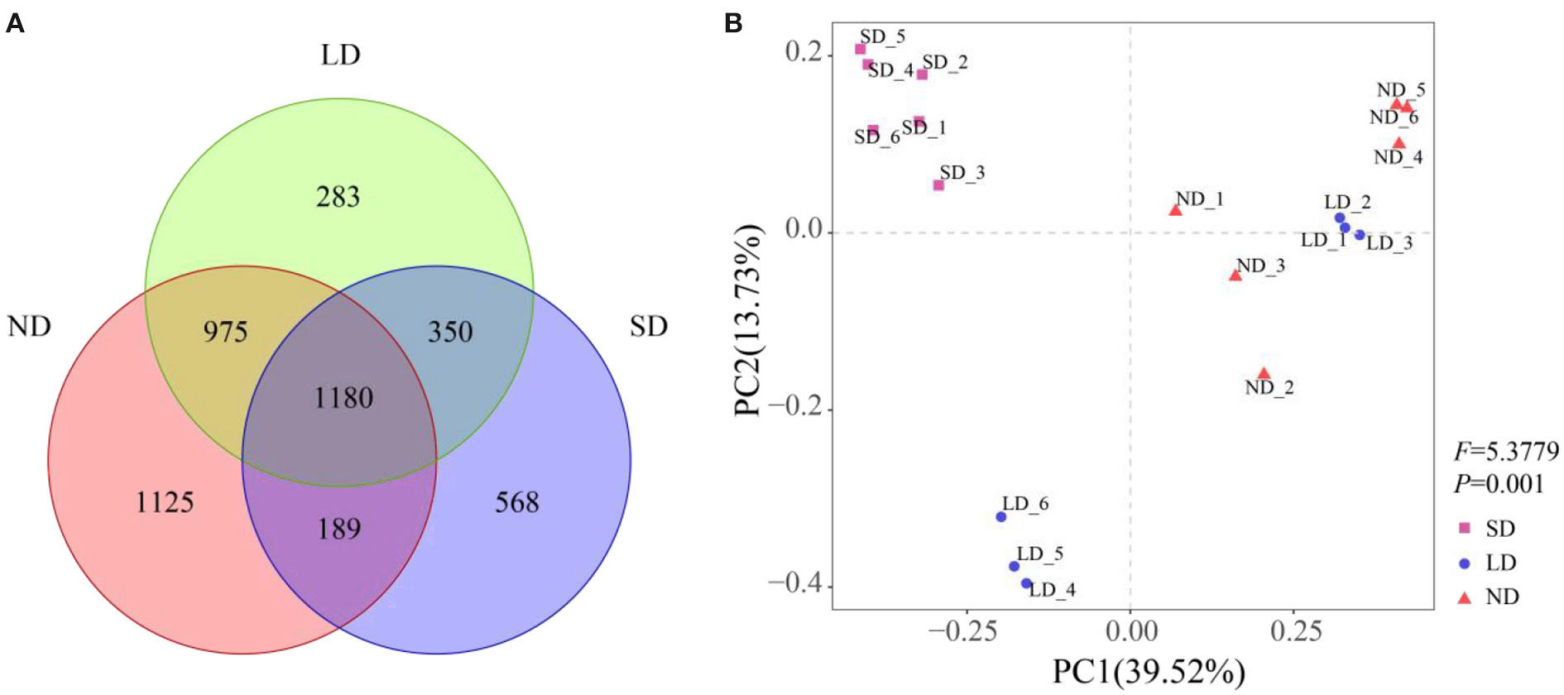
Effect of degradation on the community composition of nitrogen-fixing bacteria. **(A)** Venn diagram of different degradation stages, different colors represent different degradation stages, and overlapping areas of different colors represent intersection points (that is, common OTUs appear in areas with overlapping colors, while non-overlapping areas show unique OTUs). **(B)** Application of principal coordinate analysis (PCoA) of nitrogen-fixing bacteria community based on the Bray-Curtis different similarity matrix in different degradation stages. Different shapes represent different degradation stages. The percentage of change explained by principal coordinates is displayed on the coordinate axis, and PERMANOVA results are displayed on the right side of PCoA. ND is non-degradation and LN and SD are the light and severe degradation stages of the alpine wetland, respectively.

There were 1,180 common OTUs under different stages of degradation, accounting for 25.3% of the total OTUs. ND, LD, and SD stages had 1,125, 283, and 568 specific OTUs, respectively (Figure 3A). The PCoA results showed that nitrogen-fixing bacteria in different degradation stages gathered strongly (Figure 3B). The PERMANOVA results were consistent with the PCoA results. The PCoA could explain 39.52% of the variation in the communities containing the *nifH* gene. Among these, the first coordinate (PCoA1) distinguished the ND and SD stages, and the second coordinate (PCoA2) explained the remaining 13.73% of the variation.

**Figure 4:**
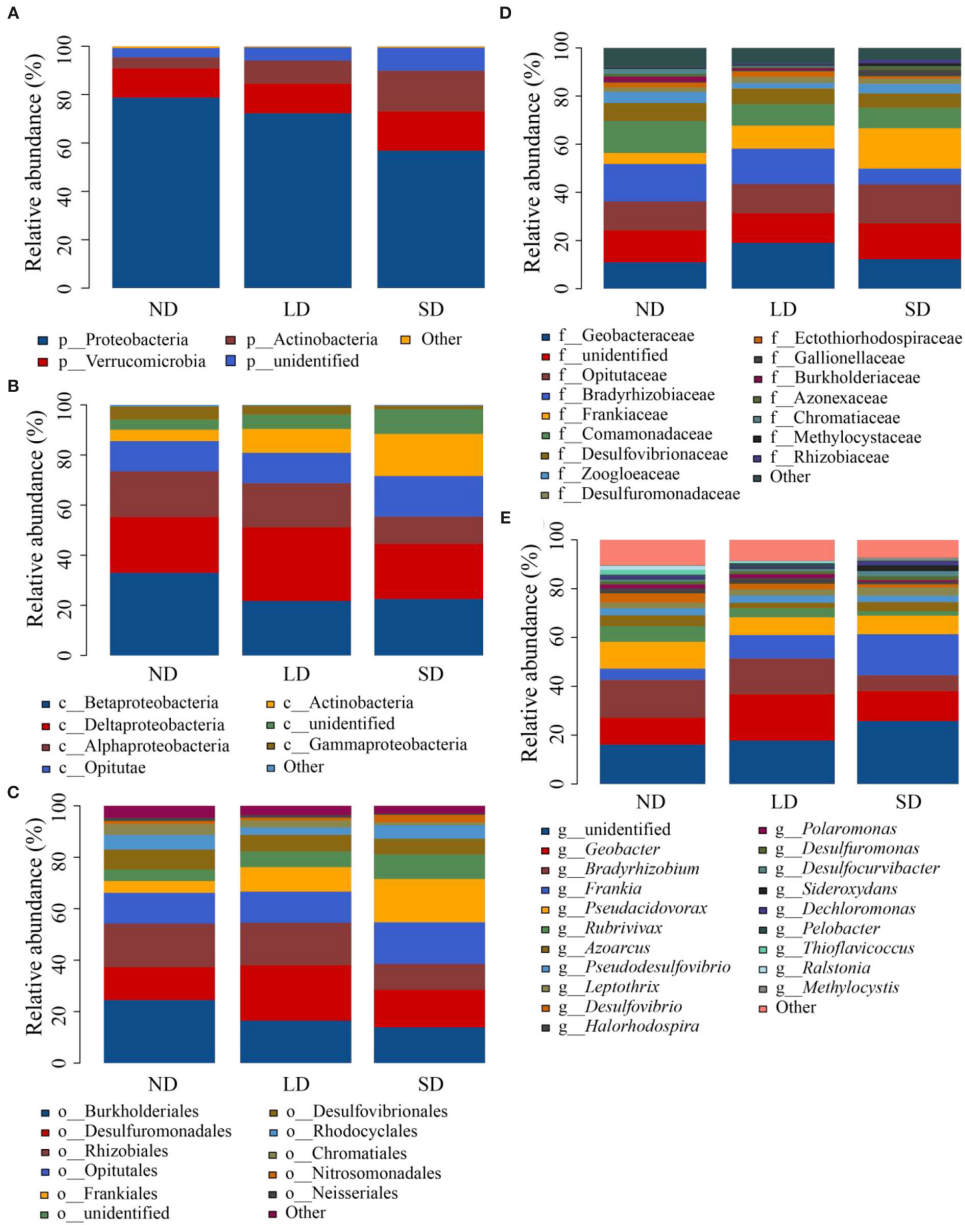
Relative abundance of phylum **(A)**, class **(B)**, order **(C)**, family **(D)**, and genus **(E)**. ND is non-degradation and LN and SD are the light and severe degradation stages of the alpine wetland, respectively.

The sequences obtained from the research were classified into 14 phyla, 19 classes, 49 orders, 95 families, and 177 genera. The proportion of major phylum, class, order, family, and genus is shown in Figure 4. The analysis of LEfSe allowed the identification of species (biomarkers) that were significantly different between groups and species or communities that significantly influenced the variability between groups. The evolution branches of nitrogen-fixing bacteria were different at various stages of degeneration. From phylum to genus, the radiating circle from inside to outside, the proportion of species with no significant difference colored yellow was small. In contrast, there were more *nifH* gene groups reflecting their differences in each degradation stage (Supplementary Figure 2), and the impact of significantly different species is shown in Supplementary Figure 3. At the ND stage of the evolutionary branching diagram, nitrogen-fixing bacteria with obvious directivity appeared at the phylum level, including Proteobacteria and Firmicutes, and Betaproteobacteria and Alphaproteobacteria at the class level, Burkholderiales and Rhizobiales at the order level, and Bradyrhizobiaceae and Burkholderiaceae at the family level. At the LD stage of the evolutionary branching diagram, nitrogen-fixing bacteria that point in a distinct direction do not appear at the phylum level, Deltaproteobacteria at the phylum level, Desulfuromonadales and Sphingomonadales at the order level and Geobacteraceae and Sphingomonadaceae at the family level. At the SD stage of the evolutionary branching diagram, the nitrogen-fixing bacteria that point clearly to the phylum level were Actinobacteria, Actinobacteria at the class level, Frankiales at the order level, and Frankiaceae and Rhizobiaceae at the family level.

**Figure 5:**
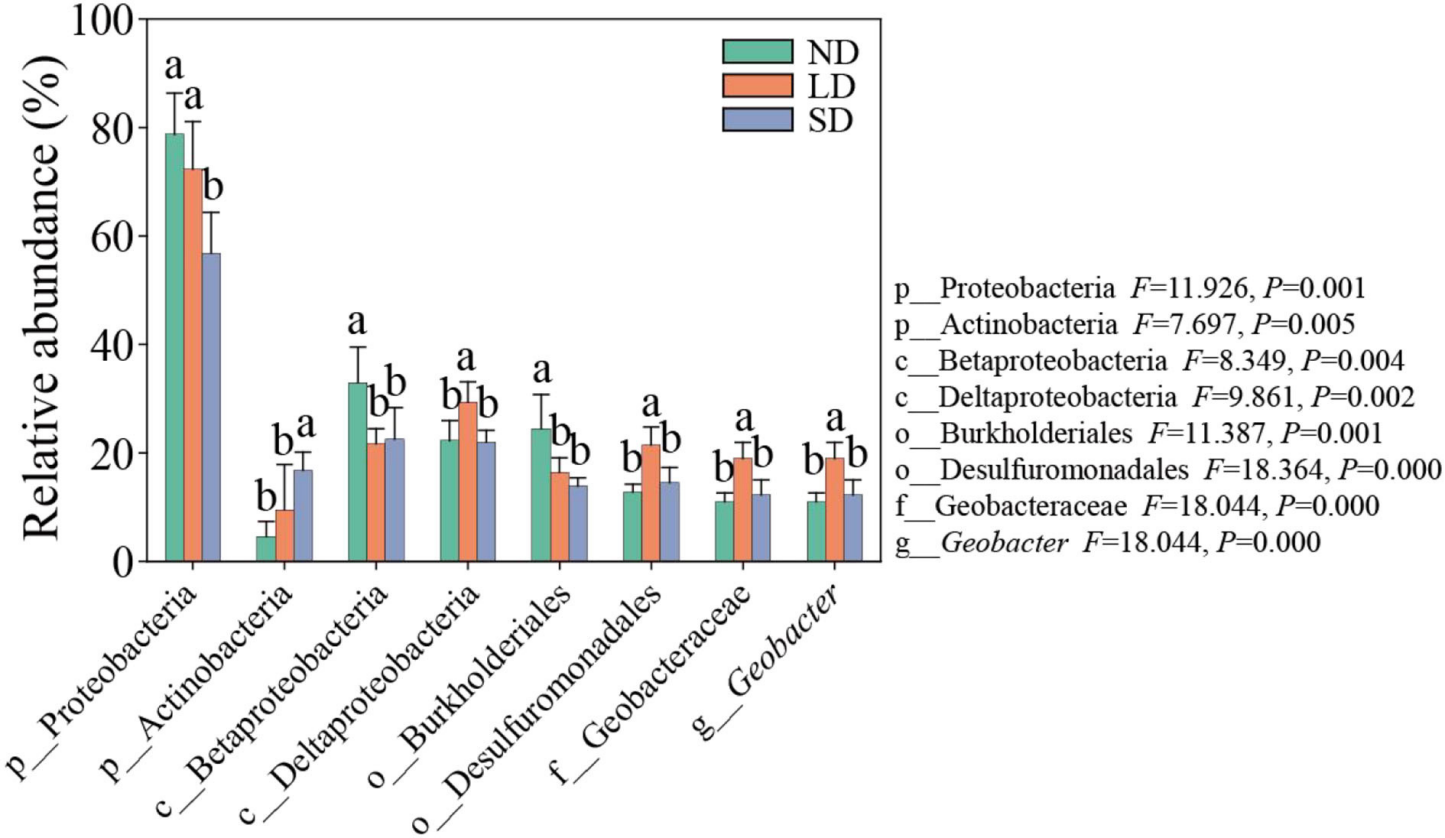
Abundance of main species of nitrogen-fixing bacteria. ND is non-degradation and LN and SD are the light and severe degradation stages of the alpine wetland, respectively. Different lowercase letters represent significant differences between different degradation stages.

The results of the ANOVA test showed significant differences in the abundance of dominant species at phylum, class, order, family, and genus levels at different stages of degradation (Figure 5). ND and LD Proteobacteria were significantly more abundant than SD. The abundance of ND and LD Actinobacteria was significantly lower than SD. ND Betaproteobacteria and Burkholderiales were significantly more abundant than LD and SD. The abundance of Deltaproteobacteria, Desulfuromonadales, Geobacteraceae, and *Geobacter* was significantly higher than that of ND and SD (*P* < 0.05). These results indicate that the degradation of the alpine wetland can significantly change the nitrogen-fixing bacteria community.

### Factors affecting nitrogen-fixing bacteria

**Figure 6:**
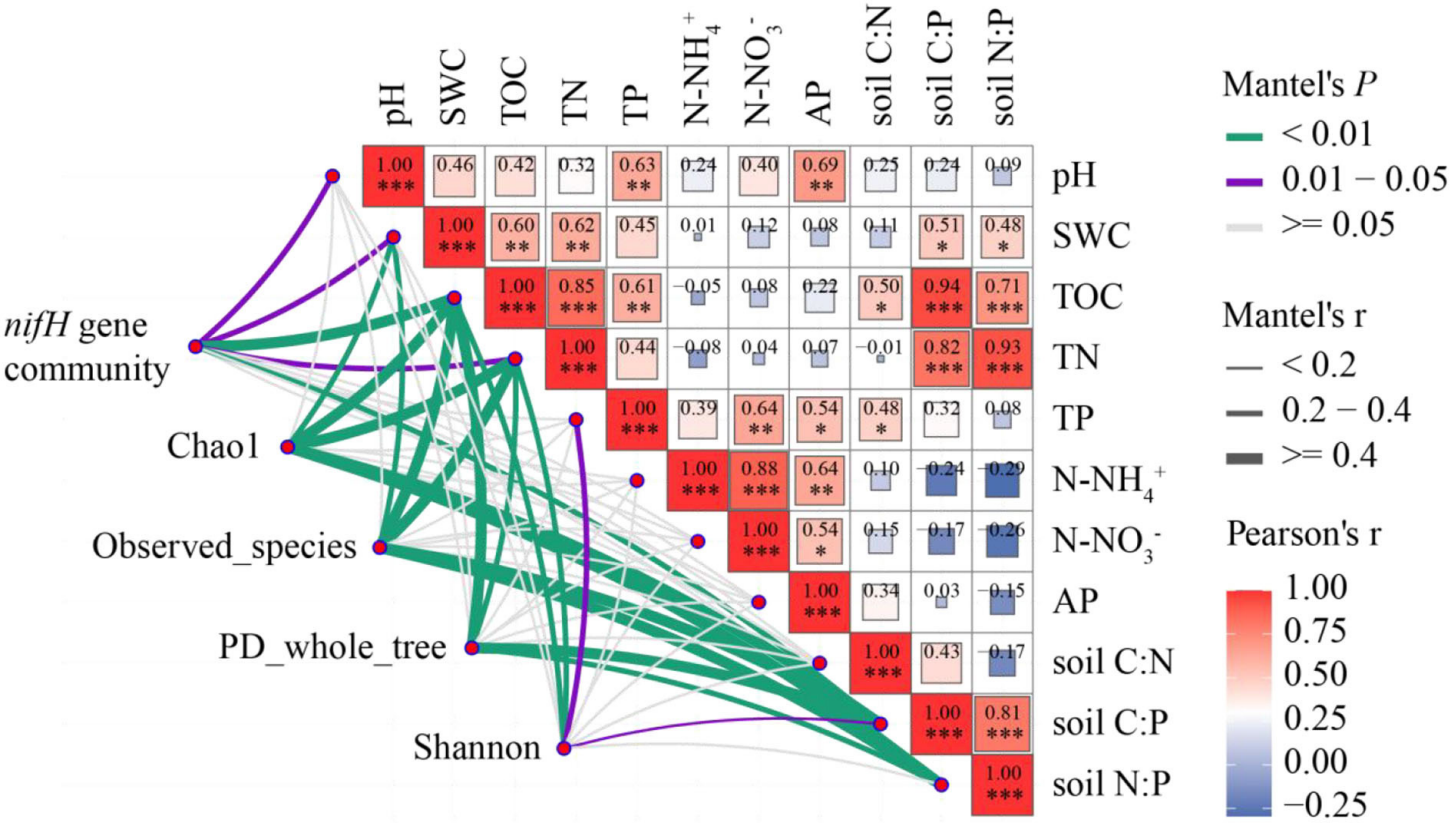
Correlations among nitrogen-fixing microbial community and diversity and soil physicochemical properties. Mantel edge width corresponds to Mantel *r*-value, and edge color indicates statistical significance. The color gradient of Pearson correlation coefficient *r* indicates the pairwise correlation of variables. The nitrogen-fixing gene community includes all nitrogen-fixing bacteria phylum. *0.01 < *P* < 0.05, **P < 0.01, ***P < 0.001.

Analysis of the Mantel test showed that pH, SWC, TOC, TN, C:P, and the abundance of nitrogen-fixing bacteria were significantly positively correlated (*P* < 0.05). SWC, TOC, TN, C:P, N:P, and Chao1 index and species number were significantly positively correlated (*P* < 0.05). TOC, TN, C:P, N:P, and PD_whole_tree diversity were significantly positively correlated (*P* < 0.05). TOC, TN, TP, C:P, and Shannon were significantly positively correlated (*P* < 0.05; Figure 6). In addition, TOC, TP, TN, and SWC were positively correlated.

**Figure 7:**
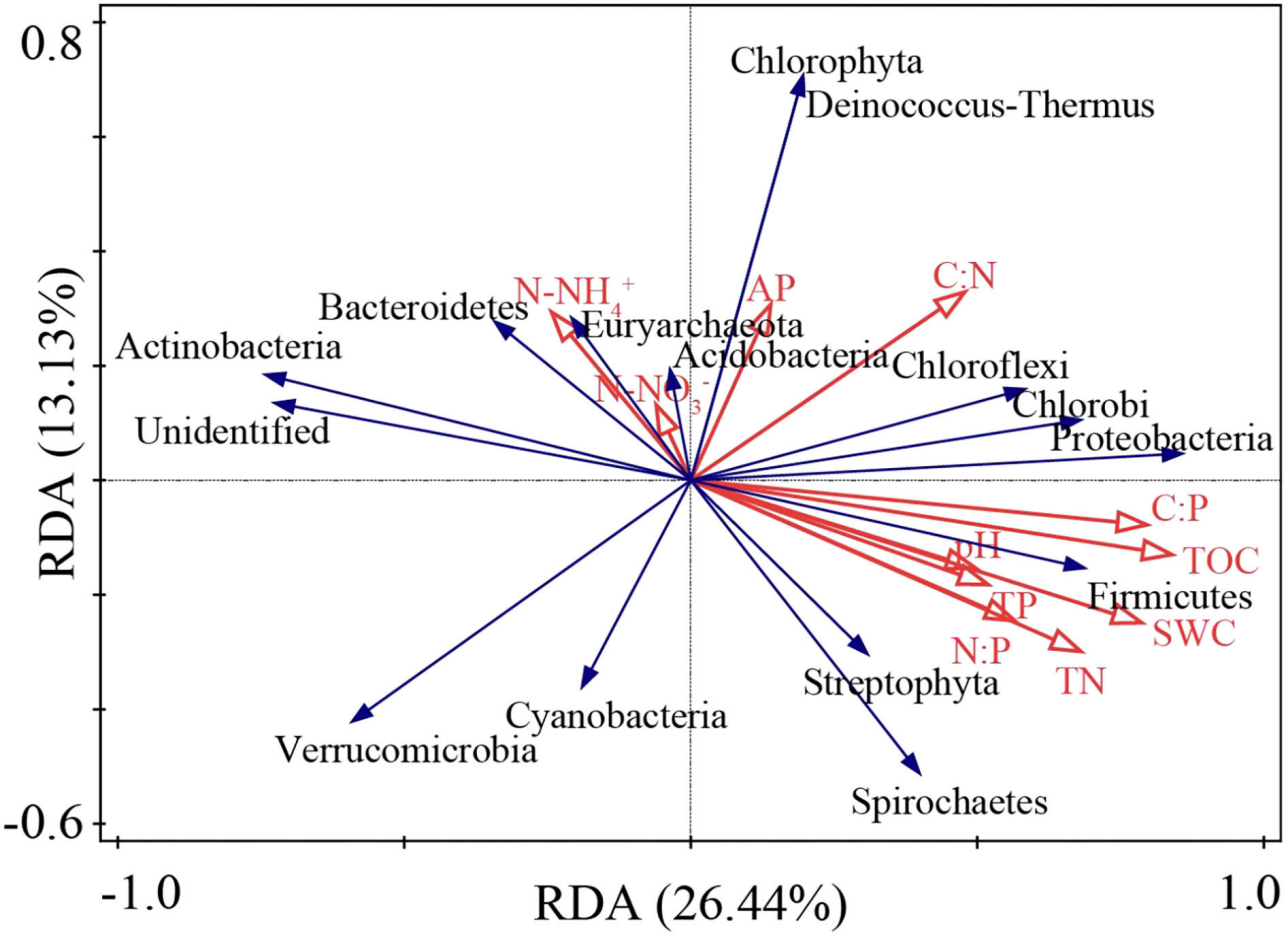
RDA ranking of soil physicochemical indexes and nitrogen-fixing bacteria abundance.

The RDA ordination results showed that the first- and second-ordination axes explained 26.44 and 13.13% of the total species variables, respectively (Figure 7). TOC, SWC, and TN accounted for 20.1, 17.4, and 15.5% of the prime index, respectively (Supplementary Table 3), as the main environmental factors influencing the abundance of the nitrogen-fixing bacteria phylum.

## Discussion

In this study, we selected different degradation stages in an alpine wetland to investigate the responses of nitrogen-fixing bacteria to the degradation severities. Our result is similar to that of Wu et al. (2016), who showed that the degradation of the alpine wetlands significantly altered the community structure of the nitrogen-fixing bacteria. It was found that the degradation of the alpine wetland changed the composition of the nitrogen-fixing bacteria community. In addition, the abundance of nitrogen-fixing bacteria was related to the change of soil properties, especially TOC, SWC, and TN were significantly related to the abundance of nitrogen-fixing bacteria community. The relative abundance of Proteobacteria, which was positively correlated with TOC, SWC, and TN, decreased significantly with the degradation of the wetland. However, the relative abundance of Actinobacteria was negatively correlated with TOC, SWC, and TN. This may be because the soil environment with good water or nutrients is beneficial to the survival and reproduction of Proteobacteria, while Actinobacteria is more suitable for poor soil. We also detected a high proportion of Verrucomicrobia in the soil of the alpine wetland, which may be attributed to its excellent survival ability in extreme environments (Che et al., 2018). However, their abundance did not change significantly during wetland degradation. There was a direct relationship between the changes of nitrogen-fixing dominant bacilli and their dominant classes, orders, families, and genera. For example, the pattern of change in Deltaproteobacteria under Proteobacteria was consistent with changes in abundance of Desulfuromonadales, Geobacteraceae, and *Geobacter*.

Zhang et al. (2020) revealed that the process of transforming a natural marsh into a meadow and then into a sandy area in Zoige on the QTP inhibits the activity of soil nitrogen-fixing bacteria. They find that degradation causes a reduction in soil water content, which in turn leads to a reduction in the rate of nitrogen fixation in wetlands through changes in plant communities, soil properties, and the composition of the nitrogen-fixing bacterial communities. In contrast, our results showed that the wetland degradation not only changes the composition of the nitrogen-fixing bacteria community, but also makes soil nitrogen-fixing bacteria more vulnerable, and then, the diversity decreases significantly with the aggravation. However, nitrogen-fixing bacteria diversity is more related to TOC, TN, TP, and soil C:P. First, soil water is widely considered to be the main limiting factor for soil microorganisms and plays a key role in building soil microbial communities (Heděnec et al., 2017; Huang et al., 2017; Noah, 2017). Second, water effectiveness is also a determinant of plant growth and vegetation community composition (Wei et al., 2017; Liu W. et al., 2018). As the underwater decreases, aquatic plants (i.e., the basic vegetation that forms the marsh soil) gradually disappear. At the same time, mesophytes (i.e., meadow vegetation) invaded and became dominant plants, while the changes in vegetation and groundwater broke the balance of soil C, N, and P (Wu et al., 2016). Zhang et al. (2020) find a negative correlation between soil TP and sedge cover, indicating little accumulation of TP as it can be rapidly used by sedge species under flooded conditions. This result also explains well the significant increase in TP when non-degradation wetland was converted to a lightly degraded wetland. In our results, light degradation of wetland reduced the above-ground biomass of Cyperaceae from 85.2 ± 16.8 g·(0.25 m^2^)^−1^ to 66.3 ± 15.5 g·(0.25 m^2^)^−1^ (Li et al., 2020). In contrast, the significant decrease in TP content from light to severe degradation was due to the strong positive correlation between TP and SOC. At this time, SOC may contribute to TP accumulation, whereas wetland degradation significantly reduces soil SOC content (Zhang et al., 2020); therefore, TP also begins to decline. Many studies have shown that phosphorus is one of the key factors limiting nitrogen fixation due to the high demand for adenosine triphosphate (ATP) by nitrogen-fixing bacteria (Vitousek et al., 2002; Reed et al., 2011). It may be the reason for the significant positive correlation between TP and nitrogen-fixing bacteria diversity. TOC is closely related to the abundance and diversity of nitrogen-fixing bacteria, which reflects the dependence of nitrogen-fixing bacteria on carbon or energy (Reed et al., 2011). The TOC per kg of soil was higher than that of TP, and the TOC of wetland degradation decreased more, so the soil C:P decreases significantly, and it also showed that the soil C:P was positively correlated with the abundance and diversity of nitrogen-fixing bacteria. In addition, TN was positively correlated with the abundance and diversity of nitrogen-fixing bacteria. First, it can be attributed to collinearity between soil nitrogen content and other soil properties (such as moisture, organic carbon, and total phosphorus), which cancels or even reverses the correlation between soil nitrogen content and nitrogen-fixing bacteria abundance (Che et al., 2018). Second, the positive correlation between soil nitrogen content and nitrogen-fixing bacteria may indicate that the community structure of nitrogen-fixing bacteria with different environmental preferences has changed due to the deterioration of the wetland soil environment. The alpine wetland soil in different stages of degradation has different nitrogen-fixing bacteria communities, these changes indicate the adaptability of microorganisms to the new environment and result in the composition of microbial communities once environmental conditions have changed (Wardle et al., 2004). The alpine wetland may be particularly sensitive to the decline of nitrogen-fixing bacteria. In turn, nitrogen inputs to degraded ecosystems will be reduced due to the reduction of soil nitrogen-fixing bacteria. The nitrogen-fixing bacteria are the main source of external nitrogen to these ecosystems, it also suggests that biological nitrogen fixation contributes significantly to the nitrogen input to the alpine wetland on the QTP. Furthermore, the first and second ordination axes of the soil nitrogen-fixing bacteria community explained 26.44% and 13.13% of the total variation in the samples, respectively. This indicates that in addition to soil properties, there are other factors that may cause changes in the community structure of nitrogen-fixing bacteria, one of which is the presence of vegetation (Sarkar, 2022). The more specific details need to be verified by further research.

The alpine wetland degenerates into the alpine meadow, and the diversity of nitrogen-fixing bacteria decreases; it is not conducive to the stability of the soil ecosystem, and it will affect the important ecological role of the alpine wetland in the process of carbon and nitrogen cycle and climate regulation. Our research results show that the degradation and succession of the alpine wetlands into alpine meadows will also lead to a significant decline in plant community productivity and ecosystem carbon sink function (Li et al., 2020). Therefore, it is very important to protect and restore the alpine wetland on the QTP. At the same time, soil microorganisms are closely related to the circulation of many elements such as soil organic matter, nitrogen, and phosphorus. Microbial processes of soil organic carbon and nitrogen transformation involve organic carbon fixation, decomposition, and transformation, organic nitrogen mineralization, nitrification, denitrification, and others. This study only involves nitrogen-fixing bacteria. The functional microorganisms involved in the carbon-nitrogen cycle also include carbon-fixing bacteria, methanogenic bacteria, methane-oxidizing bacteria, ammonia-oxidizing bacteria, denitrifying bacteria, etc. Therefore, in the future, it is necessary to further explore and analyze the driving effect of different soil functional microorganisms on carbon-nitrogen transformation and their relationship. In addition, freeze-thaw hills are typical features of the alpine wetland, which play an indicative role in the degradation of the alpine wetland. Future research should also pay attention to the changes of soil microbial communities in freeze-thaw hills and between hills during the degradation of the alpine wetland, which will be of great significance to clarify the degradation mechanism in the alpine wetland.

## Conclusion

The structure and diversity of nitrogen-fixing bacteria communities in the alpine wetlands changed by degradation severity, and the Proteobacteria were found to be the main phylum of soil nitrogen-fixing bacteria communities. In the processes of wetland degradation, the dominant phylum, class, order, family, and genus of nitrogen-fixing bacteria have changed significantly. Different soil moisture and nutrient conditions in different stages of wetland degradation strongly affected the distribution of nitrogen-fixing bacteria communities. The degradation of the alpine wetlands significantly changed the composition of nitrogen-fixing bacteria communities and reduced their diversity. TOC, SWC, and TN were identified as the main environmental factors that affect the community composition of nitrogen-fixing bacteria.

## Data availability statement

The raw sequence data from this study were deposited in the NCBI database with the study accession number: PRJNA854083 that are publicly accessible at http://www.ncbi.nlm.nih.gov/bioproject/854083.

## Author contributions

XL and CL carried out the field experiments and sampling and analyzed the data. CL wrote the original manuscript, with contributions from XL, YS, HL, and YY. All authors approved the final version of the manuscript.

## Funding

This study was financially supported by the National Natural Sciences Foundation of China (Grant No. U21A20191), the Natural Resources Investigation and Monitoring Project from central funds of Forest-grassland Ecological Protection and Restoration in the Qinghai region of Qilian Mountains National Park (Grant No. QHXH-2021-018), the 111 Project for Introducing Talents through Discipline Innovation in Colleges and Universities (D18013), and the Qinghai Science and Technology Innovation and Entrepreneurship Team Project titled Sanjiangyuan Ecological Evolution and Management Innovation Team.

## Conflict of interest

The authors declare that the research was conducted in the absence of any commercial or financial relationships that could be construed as a potential conflict of interest.

## Publisher's note

All claims expressed in this article are solely those of the authors and do not necessarily represent those of their affiliated organizations, or those of the publisher, the editors and the reviewers. Any product that may be evaluated in this article, or claim that may be made by its manufacturer, is not guaranteed or endorsed by the publisher.
