# Supplementary material for: Degradation reduces the diversity of nitrogen-fixing bacteria in the alpine wetland on the Qinghai-Tibet Plateau
Source: Front Plant Sci. 2022 Aug 4;13:939762. doi: 10.3389/fpls.2022.939762 (PMC9386517; doi:10.3389/fpls.2022.939762)
Supplement: Supplementary file 1 [file Data_Sheet_1.docx]

**Degradation reduces the diversity of nitrogen-fixing bacteria in alpine wetland on the** **Qinghai-Tibet Plateau**

Chengyi Li^1^, Xilai Li^1, 2,^ *, Yuanwu Yang^1^, Honglin Li^2^, Yan Shi^3^

^1^ College of Agriculture and Animal Husbandry, Qinghai University, Xining 810016, China.

^2^ State Key Laboratory of Plateau Ecology and Agriculture, Qinghai University, Xining 810016, China.

^3^ School of Environment, the University of Auckland, Auckland 1010, New Zealand.

* Corresponding author

E-mail address: [xilai-li@163.com](mailto:xilai-li@163.com) (X.L.Li)

**SUPPLEMENTARY MATERIAL**

**1.1 Supplementary Figures**


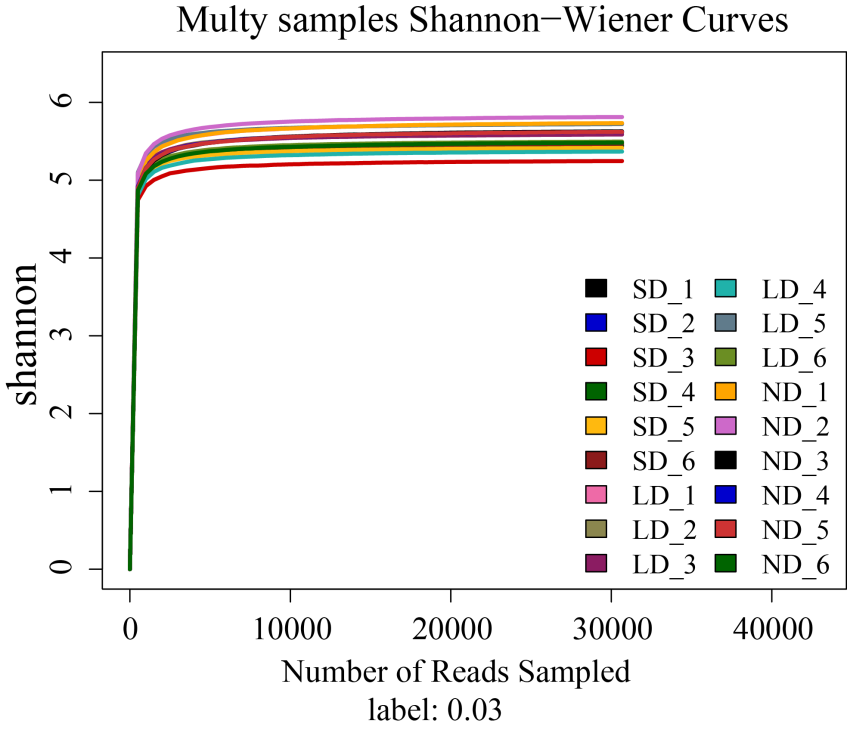


**SUPPLEMENTARY** **FIGURE 1 Shannon-Wiener curve**

**Note:** ND is non-degradation, LN and SD are the light and severe degradation stages of alpine wetland respectively. 1, 2, 3, 4, 5, 6 represent six replications of the same treatment.


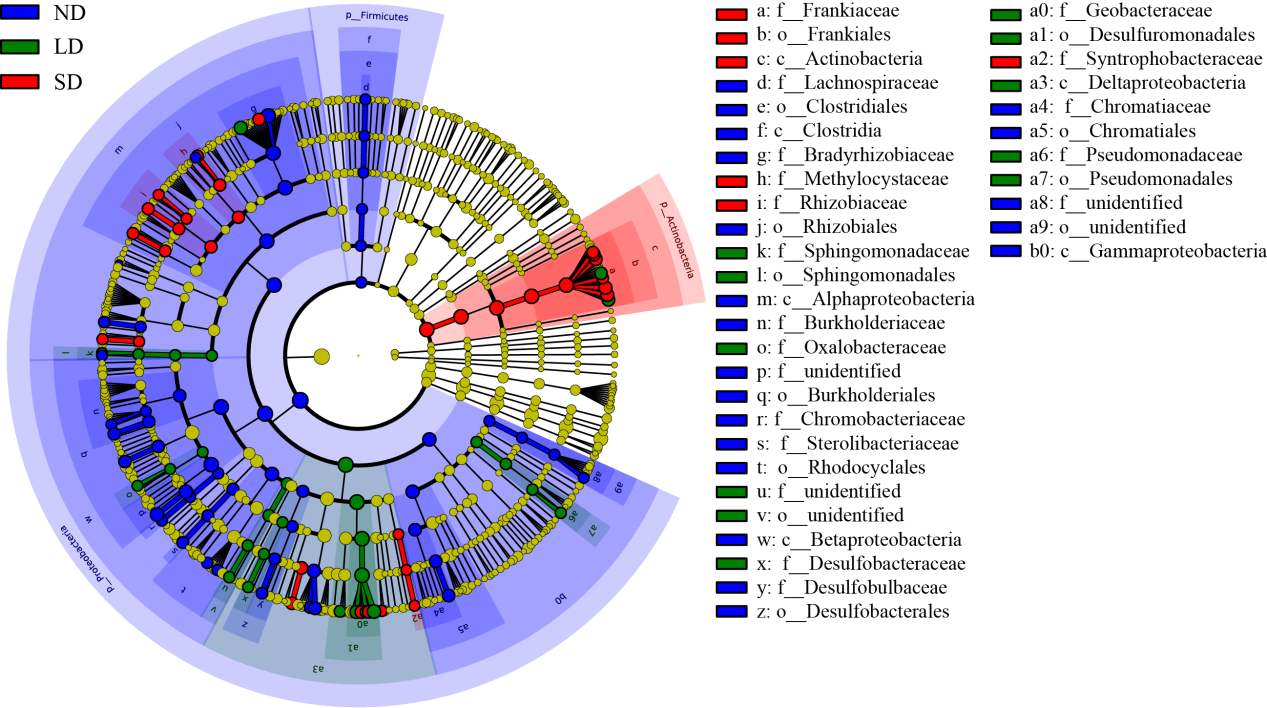


**SUPPLEMENTARY** **FIGURE 2** Evolutionary branch diagram of LEfSe gene of *nifH*

**Note:** ND is non-degradation, LN and SD are the light and severe degradation stages of alpine wetland respectively.


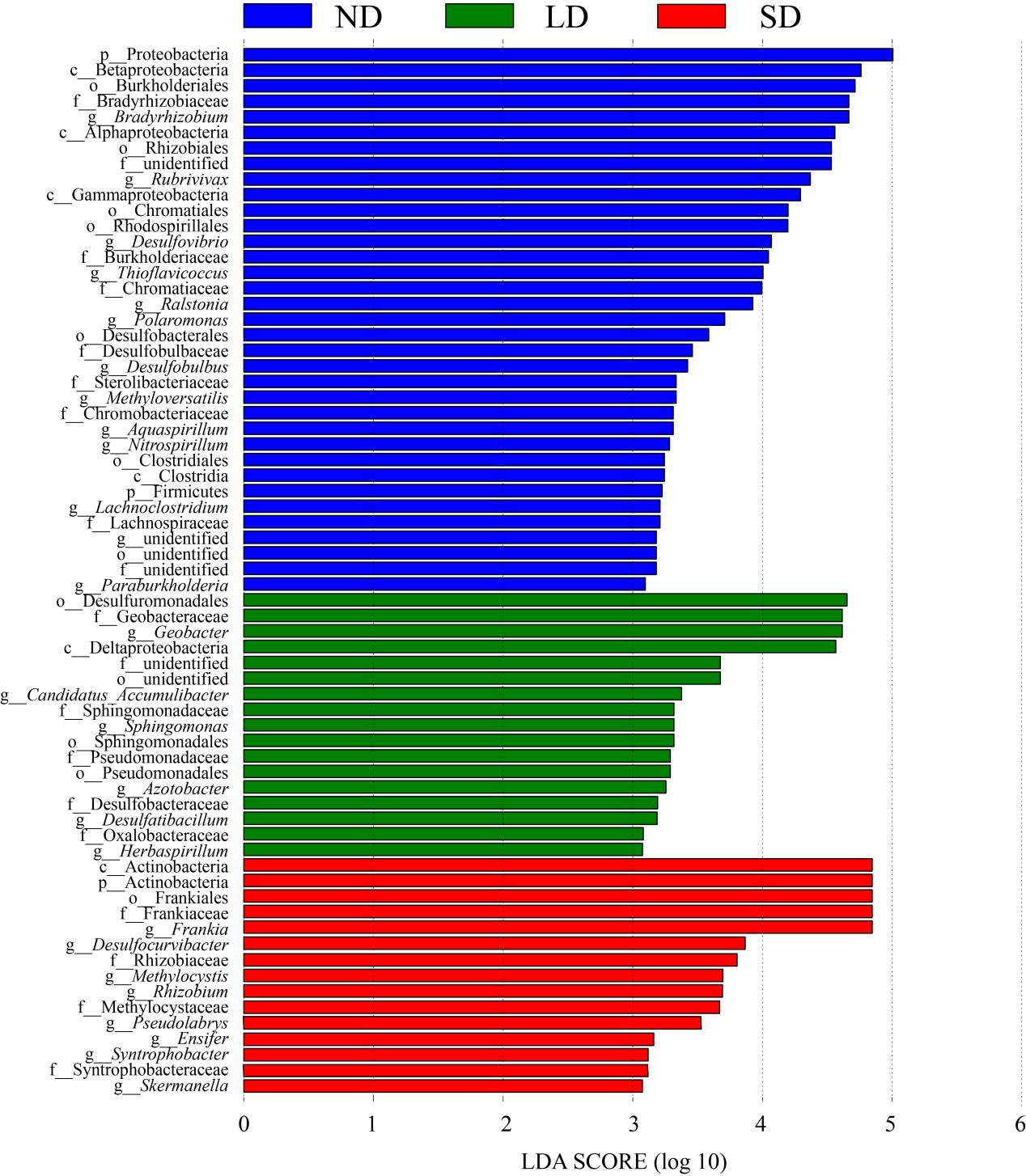


**SUPPLEMENTARY** **FIGURE 3** Linear discriminant analysis (LDA) of markers (score > 3)

**Note:** ND is non-degradation, LN and SD are the light and severe degradation stages of alpine wetland respectively.

**1.2 Supplementary Tables**

**SUPPLEMENTARY** **TABLE 1** Different degradation stages and land features

| Degeneration stage | Dominant plant species | Coverage/% | Height/cm | Amount of freeze-thaw mounds | Size of freeze-thaw mounds/m^2^ |
| --- | --- | --- | --- | --- | --- |
| Non-degradation | *Kobresia tibetica, Carex moorcroftii* | 98 | 16.21 | 5±1 | 0.10±0.02 |
| Light degradation | *Kobresia tibetica, Kobresia humilis, Carex moorcroftii* | 88 | 12.42 | 10±1 | 0.02±0.01 |
| Severe degradation | *Kobresia humilis,*  *Poa crymophila* | 60 | 7.45 | — | — |

**SUPPLEMENTARY** **TABLE 2** Physical and chemical indexes of soil samples in different degradation stages of alpine wetland

| Index | Degree of degeneration | | | *F*-value | *P*-value |
| --- | --- | --- | --- | --- | --- |
|  | Non-degradation | Light degradation | Severe degradation |  |  |
| pH | 7.61±0.09a | 7.75±0.09a | 7.24±0.45b | 5.703 | 0.014 |
| SWC (%) | 63.40±4.15a | 58.72±1.07b | 55.95±2.58b | 10.183 | 0.002 |
| TOC (g·kg^-1^) | 116.75±9.44a | 96.95±7.13b | 58.70±9.26c | 69.482 | 0.000 |
| TN (g·kg^-1^) | 9.28±1.19a | 6.94±0.65b | 5.14±0.38c | 39.123 | 0.000 |
| TP (g·kg^-1^) | 0.44±0.02b | 0.8±0.02a | 0.38±0.03c | 26.768 | 0.000 |
| N-NH_4_^+^ (mg·kg^-1^) | 13.82±5.40a | 18.34±3.75a | 15.50±2.32a | 1.936 | 0.179 |
| N-NO_3_^-^ (mg·kg^-1^) | 3.27±1.30b | 4.96±0.82a | 3.29±0.58b | 6.258 | 0.011 |
| AP (mg·kg^-1^) | 20.95±2.56ab | 24.95±4.15a | 19.91±3.21b | 3.735 | 0.048 |
| Soil C:N | 12.73±1.76a | 14.12±2.01a | 11.41±1.62a | 3.284 | 0.066 |
| Soil C:P | 265.59±23.02a | 202.87±15.66b | 155.57±29.55c | 33.304 | 0.000 |
| Soil N:P | 21.06±2.29a | 14.52±1.43b | 13.65±1.84b | 27.200 | 0.000 |

**Note:** pH: pH; SWC: soil water content; TOC: soil organic carbon; TN: total nitrogen; TP: total phosphorus; N-NO_3_^-^: nitrate nitrogen; N-NH_4_^+^: ammonia nitrogen; AP: available phosphorus; Soil C:N represents the ratio of organic carbon to total nitrogen; Soil C:P represents the ratio of organic carbon to total phosphorus; Soil N:P represents the ratio of total nitrogen to total phosphorus. Different lowercase letters represent significant differences between different degradation stages.

**SUPPLEMENTARY TABLE 3** Simple Effects Results

| Name | Explains % | pseudo-*F* | *P*-value |
| --- | --- | --- | --- |
| TOC | 20.1 | 4.0 | 0.002 |
| Soil C:P | 18.4 | 3.6 | 0.002 |
| SWC | 17.4 | 3.4 | 0.006 |
| TN | 15.5 | 2.9 | 0.004 |
| TP | 12.6 | 2.3 | 0.012 |
| Soil N:P | 12.5 | 2.3 | 0.028 |
| pH | 10.0 | 1.8 | 0.082 |
| Soil C:N | 8.9 | 1.6 | 0.110 |
| AP | 8.0 | 1.4 | 0.146 |
| N-NH_4_^+^ | 6.1 | 1.0 | 0.376 |
| N-NO_3_^-^ | 4.4 | 0.7 | 0.728 |
